# Declining life expectancy in the Great Lakes region: contributors to Black and white longevity change across educational attainment

**DOI:** 10.1186/s12889-023-15668-x

**Published:** 2023-04-26

**Authors:** Max Tyler Roberts, Sojung Lim, Eric N. Reither

**Affiliations:** 1 Washington, USA; 2 Utah State University, 0730 Old Main Hill, Logan, UT 84322 USA

**Keywords:** Life expectancy, Longevity, Racial disparities, Great lakes, Cause of death

## Abstract

**Background:**

The East North Central Census division (aka the Great Lakes region) experienced a decrease in life expectancy of 0.3 years from 2014 to 2016 – one of the largest declines across the nine Census divisions. Disadvantaged groups that typically have below-average life expectancy, including Black individuals and those without a college education, may have been disproportionately affected by this longevity shift. This investigation examines life expectancy changes among different sex, race, and education groups in the Great Lakes region, and how specific causes of death contributed to within-group longevity changes over time and across age.

**Methods:**

We used 2008 to 2017 death counts from the National Center for Health Statistics and American Community Survey population estimates to measure within-group change in life expectancy at age 25 among non-Hispanic Black and white males and females by educational attainment. We decomposed life expectancy change over time for each subgroup by 24 causes of death and measured their contribution to longevity change across 13 age groups.

**Results:**

Among persons with ≤ 12 years of education, white males and females experienced 1.3- and 1.7-year longevity declines respectively, compared to a 0.6-year decline among Black males and a 0.3-year decline among Black females. Life expectancy declined among all groups with 13–15 years of education, but especially Black females, who experienced a 2.2-year loss. With the exception of Black males, all groups with 16 + years of education experienced longevity gains. Homicide contributed 0.34 years to longevity decline among Black males with ≤ 12 years of education. Drug poisoning made large contributions to longevity losses among Black females with ≤ 12 years of education (0.31 years), white males and females with 13–15 years of education (0.35 and 0.21 years, respectively), and white males and females with ≤ 12 years of education (0.92 and 0.65 years, respectively).

**Conclusions:**

Public health efforts to reduce the risks of homicide among Black males without a college education and drug poisoning among all groups could improve life expectancy and reduce racial and educational longevity disparities in the Great Lakes region.

**Supplementary Information:**

The online version contains supplementary material available at 10.1186/s12889-023-15668-x.

## Background

**Fig. 1:**
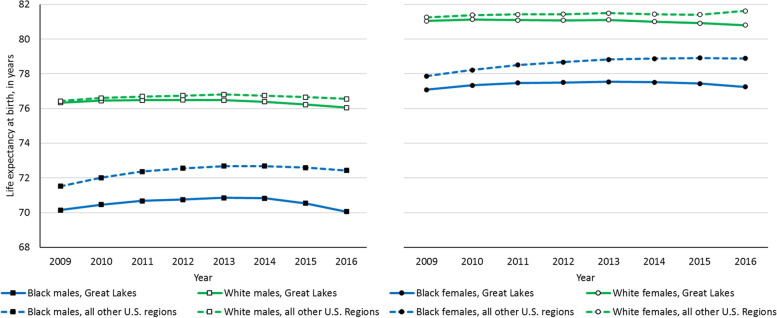
Life expectancy at birth trends in the Great Lakes region and all other U.S. regions for non-Hispanic Black and non-Hispanic white males and females

Life expectancy in the United States recently declined for the first time since 1993, with losses occurring in three consecutive years from 2014 to 2016 [1–6]. With the exception of the West South Central Census division, all Census divisions experienced longevity losses during this time. Of special interest to this study is the East North Central Census division, composed of Illinois, Indiana, Michigan, Ohio, and Wisconsin (hereafter, the Great Lakes region), which experienced a 0.3-year longevity decline – one of the largest life expectancy losses across the nine Census divisions of the U.S.[1]. Moreover, longevity decline generally began sooner in the Great Lakes region than other U.S. regions, widening disparities between major demographic groups, especially non-Hispanic Black people (Fig. 1).

The recent longevity decline observed in the Great Lakes region may have disproportionately affected Black adults and people without a college education, as these groups typically have below-average life expectancy [7]. Existing research has shown this pattern to be true at the national level, with low-education groups and Black males and females experiencing decreasing life expectancy in recent years [8], but there are no systematic analyses of contributors to longevity decline across different subgroups in the Great Lakes region. This investigation seeks to determine what causes of death contributed most to within-group longevity change in the Great Lakes region, with a focus on differences by sex, race, and educational attainment.

The education gradient in life expectancy is well documented in the U.S. [8–13], such that more education generally translates into greater longevity [11, 14, 15]. Education differences in life expectancy have grown substantially over time, with life expectancy among college-educated groups diverging sharply from those with a high school education or less. In 2017, those with a 4-year college degree or higher could expect to live nearly 10 years longer than those with a high school diploma or less [8]. Those without a college education are at greater risk of preventable causes of death that are influenced by behaviors such as homicide, accidents, and smoking-related illnesses [10, 16]. A recent study found that the mortality rate for preventable causes of death was 90% greater among U.S. adults without a high school diploma between the ages of 45 and 64, compared to those with a college degree [16]. Increased rates of drug poisoning (i.e., drug overdose) has been a key driver of recent declines in life expectancy in the U.S. [2, 8, 14, 16]. There is evidence to suggest that drug poisoning has struck non-Hispanic white people particularly hard in recent years, as well as those without a college education, for whom drug poisoning accounted for one-fifth of all deaths in 2011 [14]. Other causes of death including heart disease, cancer, and chronic obstructive pulmonary disease (COPD) have been found to be key contributors to widening differences in mortality across educational groups [9].

Life expectancy disparities in the U.S. also persist across race groups, with white people consistently outliving their Black counterparts [17–21]. In 2009, white males in the Great Lakes region outlived Black males by an average of 6.7 years, and white females outlived Black females by an average of 4.7 years [22]. Evidence from a study of Wisconsin suggests that this is partly due to disproportionate mortality from homicide among young Black males, and disproportionate mortality from heart disease and cancer among older Black males and females [23]. Ongoing racial longevity disparities merit further investigation, particularly at a place and time where the average life expectancy is generally in a state of decline.

Although informative, existing research explaining educational longevity disparities in the U.S. is limited, including in the Great Lakes region. To date, few studies have analyzed mortality and longevity outcomes in the Great Lakes region [1, 22, 24] – mostly analyzing the region as part of a larger study of the U.S. While these studies have documented racial longevity disparities, noting the impact of some broad causes of death on mortality outcomes, they have not measured the contribution of specific causes of death to racial and educational longevity disparities. A recent study measured the contribution of select causes of death to change in life expectancy at age 25 for Black and white males and females in the U.S. by educational attainment [8]. The study found that life expectancy decreased among those without a college education, regardless of race, and that increased drug poisoning among those with a high school education or less likely contributed to widening educational differences in adult life expectancy. The current study builds on prior research and focuses on the Great Lakes region as it is important to continue to examine the contribution of specific causes of death to longevity outcomes so that targeted interventions can be successful in reducing mortality risks, improving longevity, and eliminating disparities.

This investigation will address longevity change in the Great Lakes region by pursuing three aims. First, we will trace life expectancy at age 25 (the age at which adults generally have completed their formal education) among non-Hispanic Black and white males and females by educational attainment from 2008 to 2017 to determine how life expectancy has changed over time for different subgroups. Second, we will decompose within-group change in life expectancy at age 25 from 2008 to 2017 into specific causes of death across three different levels of educational attainment (i.e., ≤ 12, 13–15, 16 + years of education) to determine which causes contributed most to longevity change among Black and white males and females. Third, we will assess the contribution of specific causes of death for 13 age groups to identify where across the life course each cause contributed most to within-group change in life expectancy. Results from this investigation can inform public health stakeholders to help guide policies and programs to improve longevity disparities among disadvantaged groups in the Great Lakes region.

## Methods

### Data

Data for this investigation were acquired from the National Center for Health Statistics (NCHS) and included 2008–2017 restricted-access multiple cause of death all-county micro data files [25]. These restricted files provided uncensored death counts by period of observation, age, sex, race, ethnicity, educational attainment, and cause of death. We used NCHS death counts (*D*_*ij*_) and American Community Survey population estimates (*N*_*ij*_) to calculate death rates (*M*_*ij*_ = *D*_*i*_/*N*_*ij*_) for each subgroup (*i*) and period of observation (*j*) in the Great Lakes region. Following previous work [7], we used American Community Survey 1-year, person-weighted population estimates as denominators, which were available by the same demographic characteristics as NCHS data [26].

### Measures

This investigation focused on non-Hispanic white people and non-Hispanic Black people as they were the two largest race groups in the Great Lakes region, making up 80% and 12% of the total population, respectively [27]. Due to the details of the analyses in this study (e.g., the number of deaths from hypertension in 2016 among non-Hispanic Black females ages 45–49 with 13–15 years of education), other race groups were excluded from analyses as small counts would produce insufficient data. We stratified the analysis by sex, as males and females tend to experience differing mortality regimes [28, 29]. The investigation therefore focused on four race-sex groups: non-Hispanic Black females, non-Hispanic Black males, non-Hispanic white females, and non-Hispanic white males (hereafter, Black females, Black males, white females, and white males).

We categorized age 25 years and older into 13 different groups which included a series of five-year age intervals from 25–29 to 80–84, and an open-ended category for ages 85 and older. Following previous work, we categorized educational attainment into three groups: ≤ 12 years, 13–15 years, and 16 + years of education [8]. Those with ≤ 12 years of education included anyone with a high school diploma, GED, or less education. Those with 13–15 years of education included anyone who had completed one, two, or three years of college, or had an Associate degree but did not have a 4-year college degree. Lastly, those with 16 + years of education included anyone that had 4 years of college education or more, or had a Bachelor’s, Master’s, or Doctorate degree.

We included 24 sex-specific causes of death in our analysis that were leading causes of death among Black people and white people, as well as causes of death that contributed to education-related mortality disparities [10, 21, 30]. These causes of death included: Alzheimer’s disease, breast cancer, colorectal cancer, esophageal cancer, liver cancer, lung cancer, pancreatic cancer, prostate cancer (males only), all other cancers, chronic lower respiratory disease, cerebrovascular disease, diabetes, heart disease, HIV, homicide, hypertension, influenza and pneumonia, liver disease, nephritis, septicemia, suicide, drug poisoning, motor vehicle accidents, all other unintentional injuries, and a residual category for all remaining causes of death. We disaggregated all cancers into seven specific types of cancer, as cancers differ in their etiology, prevention, and treatment. Additionally, we disaggregated unintentional injuries into drug poisoning and motor vehicle accidents, as these are the two leading forms of accidental death which require different public health interventions [31]. All causes of death were categorized in accordance with the International Classification of Disease, Tenth Revision (ICD-10) [32], and were coded using the Department of Vital Statistics 358-recode [33]. A complete coding scheme of all causes of death for the study can be found in the additional files (see Additional file 1).

### Analysis

Analyses for this study were conducted using Microsoft Excel 2016 [34]. Using data from 2008 to 2017, we aggregated three years of death counts (*D*_*ij*_) and population estimates (*N*_*ij*_) into single cross-sectional periods of observation spanning from 2009 (i.e., 2008 to 2010) to 2016 (i.e., 2015 to 2017) to reduce random year-to-year fluctuations in mortality rates (*M*_*ij*_).

To address the first aim of the investigation (i.e., determine how life expectancy has changed over time in the Great Lakes region by sex, race, and educational attainment), we employed period life table analysis to calculate life expectancy at age 25 for each race-sex-education group (e.g., Black females with 16 + years of education). Following previous work, we analyzed life expectancy at age 25 (*e*_*25*_) as this is the age at which most adults have completed their education [8, 9]. We employed iterative graduation techniques to the life tables to estimate the average person years lived (_*n*_*a*_*x*_) for persons living from age *x* to *x* + *n*. Results from this analysis helped generate visualizations of changing life expectancy at age 25 from 2009 to 2016 in the Great Lakes region across different sex-race-education groups.

We employed Arriaga’s decomposition methods to address the second and third aims of the investigation, following previous work [11, 23, 35]. Decomposition methods reveal important contributors to overall differences in life expectancy between groups or changes in life expectancy within groups. In this study, we decomposed changes in life expectancy from 2009 to 2016 for twelve groups categorized by race, sex, and education (e.g., Black males with ≤ 12 years of education). Readers interested in a detailed description of mortality decomposition methods should consult Arriaga, 1984 [36] and 1989 [37]. This approach allowed us to disaggregate the total change in life expectancy from 2009 to 2016 into smaller portions attributable to specific causes of death. Specifically, to address the second aim of the investigation (i.e., determine which causes of death contributed most to life expectancy change), we decomposed the change in life expectancy from 2009 to 2016 into portions attributable to 24 causes of death for each subgroup. To address the third aim of the investigation (i.e., identify where across the life course each cause of death contributed most to change in life expectancy), we calculated the total contribution, in years, of each cause of death across 13 age categories for each subgroup.

## Results

Table 1 displays age-standardized mortality rates and education prevalence among Black and white males and females ages 25 and older in the Great Lakes region in 2016. Black males in the Great Lakes region had a higher overall mortality rate (1,752.8 per 100,000 population) than white males (1,347.7 per 100,000 population), and Black females had a higher overall mortality rate (1,158.2 per 100,000 population) than white females (976.2 per 100,000 population). With respect to educational attainment, white males and females had higher levels of education than their Black counterparts. Whereas more than 30% of both white males and females had 16 + years of education, only 20% of Black females and 15% of Black males had 16 + years of education.

**Table 1:** Age-standardized mortality rates and education prevalence among Black and white people ages 25 + by sex, race/ethnicity, and education in the Great Lakes region, 2016

	**non-Hispanic Black**	**non-Hispanic white**
**Mortality rate**		
**Sex**		
Male	1,752.8	1,347.7
Female	1,158.2	976.2
**Education**		
< = 12		
Male	2,185.5	1,856.2
Female	1,435.4	1,302.6
13–15		
Male	1,236.0	987.1
Female	974.3	704.0
16 +		
Male	1,153.5	882.8
Female	799.6	654.8
**Education prevalence**		
< = 12		
Male	51.4%	39.7%
Female	41.3%	37.7%
13–15		
Male	33.3%	29.6%
Female	38.2%	31.0%
16 +		
Male	15.3%	30.7%
Female	20.5%	31.3%

Mortality rates are age-standardized to the 2000 U.S. population and expressed per 100,000 population

Black and white males with ≤ 12 years of education faced mortality rates of 2,185.5 and 1,856.2 deaths per 100,000 population, respectively, which were about two times greater than the mortality rates of their counterparts with 13–15 and 16 + years of education. Black females with ≤ 12 years of education faced a mortality rate of 1,435.4 deaths per 100,000 population, which was also two times greater than mortality rates among Black females with 16 + years of education. The mortality rate among white females with ≤ 12 years of education (1,302.6 deaths per 100,000 population) was about two times greater than the mortality rate among white females with 13–15 years of education (704.0 deaths per 100,000 population) and 16 + years of education (654.8 deaths per 100,000 population).

### Aim 1: Determine how life expectancy at age 25 has changed over time in the Great Lakes region by sex, race, and educational attainment

**Fig. 2:**
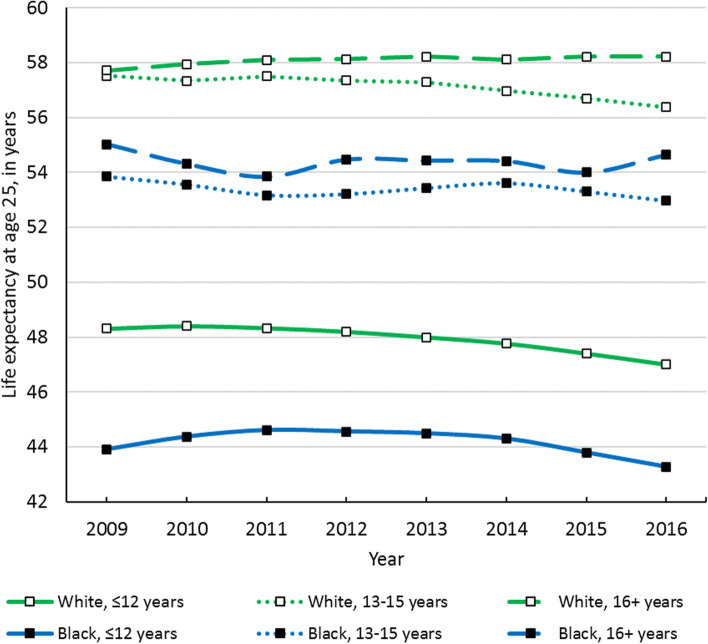
Life expectancy at age 25 over time of Black and white males in the Great Lakes region by education

**Fig. 3:**
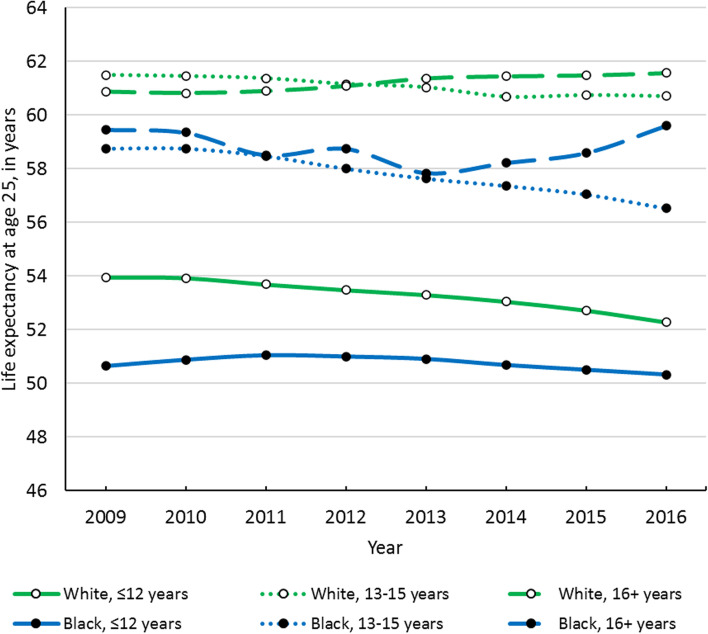
Life expectancy at age 25 over time of Black and white females in the Great Lakes region by education

Although national life expectancy declined in 2015, some groups in the Great Lakes region experienced this decline much earlier. Longevity decline started in 2011 among white males with ≤ 12 years of education and in 2012 among white males with 13–15 years of education (see Fig. 2). White females with ≤ 12 years of education began to experience life expectancy decline in 2010, while white females with 13–15 years of education experienced declines starting in 2011 (see Fig. 3). Life expectancy among Black males and females with ≤ 12 years of education began to decline in 2013 and 2012, respectively, while Black females with 13–15 years of education experienced longevity decline in 2011 (see Figs. 2 & 3). In contrast, the highest-educated white males, white females, and Black females all experienced steady increases in life expectancy over the study period (see Figs. 2 & 3).

From 2009 to 2016, white males with ≤ 12 and 13–15 years of education experienced 1.3- and 1.1-year declines in life expectancy, respectively, which were the largest longevity declines among all males (see Table 2). However, white males with 16 + years of education experienced a longevity increase of more than half a year from 2009 to 2016. Meanwhile, all Black males experienced longevity declines over the study period. Life expectancy declined by nearly one year among Black males with 13–15 years of education and by over half a year among Black males with ≤ 12 years of education. Black males with 16 + years of education experienced a 0.3-year decline in their life expectancy.

**Table 2:** Life expectancy at age 25 in the Great Lakes region by year, sex, race/ethnicity, and education

	**Black life expectancy**		**White life expectancy**	
	**Males**	**Females**	**Males**	**Females**
** ≤ 12 years**				
2009	43.91	50.65	48.31	53.96
2016	43.27	50.33	47.01	52.28
Total change	-0.64	-0.32	-1.30	-1.68
**13–15 years**				
2009	53.85	58.76	57.51	61.49
2016	52.96	56.53	56.38	60.71
Total change	-0.89	-2.23	-1.13	-0.78
**16 + years**				
2009	55.02	59.46	57.71	60.87
2016	54.71	59.68	58.31	61.67
Total change	-0.31	0.22	0.60	0.80

Similar to males, white females with ≤ 12 years of education experienced a large decline in life expectancy of 1.7 years from 2009 to 2016, while white females with 13–15 years of education saw their life expectancy decline by 0.8 years (see Table 2). White females with 16 + years of education appreciated a 0.8-year increase in life expectancy. Black females with 16 + years of education also experienced a small longevity increase of 0.2 years over the study period. Conversely, Black females with ≤ 12 years of education saw their life expectancy decline by 0.3 years, and Black females with 13–15 years of education experienced the greatest life expectancy decline of all race-sex-education groups (2.2 years).

### Aim 2: Determine which causes of death contributed most to change in life expectancy across educational attainment over time

Homicide was responsible for a 0.34-year decline in life expectancy from 2009 to 2016 among Black males with ≤ 12 years of education, followed by drug poisoning which contributed 0.25 years to longevity decline (see Table 3). Similarly, drug poisoning contributed 0.30 years to longevity decline among Black males with 13–15 years of education, followed by homicide which contributed 0.13 years to longevity decline. Among Black males with 16 + years of education, heart disease contributed 0.12 years to longevity decline.

**Table 3:**
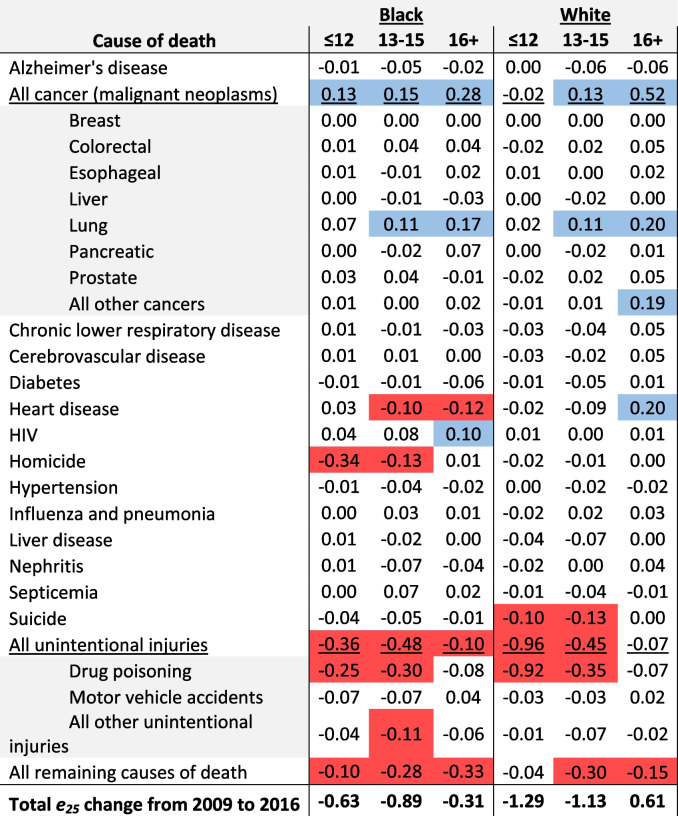
Contribution, in years, of specific causes of death to change in life expectancy from 2009 to 2016 among Black and white males in the Great Lakes region by education

Values represent the contribution, in years, to change in life expectancy from 2009 to 2016. Negative values reflect life expectancy losses over time. Conversely, positive values reflect life expectancy gains over time. Cells in red represent contributions less than or equal to -0.10 and cells in blue represent contributions greater than or equal to 0.10

Among white males, drug poisoning contributed most to longevity declines across all education levels, but especially among those with ≤ 12 years of education, where it contributed 0.92 years to longevity decline. Drug poisoning contributed 0.35 years to longevity decline among white males with 13–15 years of education and 0.07 years to longevity decline among those with 16 + years of education. Suicide was another notable contributor to longevity decline among white males, particularly among those with ≤ 12 and 13–15 years of education, where it contributed 0.10 and 0.13 years, respectively.

Among Black females with ≤ 12 years of education, drug poisoning contributed most to longevity decline over the study period, contributing 0.31 years to the overall decline (see Table 4). Heart disease contributed 0.50 years to longevity decline among Black females with 13–15 years of education. Alzheimer’s disease contributed 0.34 years to longevity decline among Black females with 16 + years of education, and a 0.31-year decline among those with 13–15 years of education.

**Table 4:**
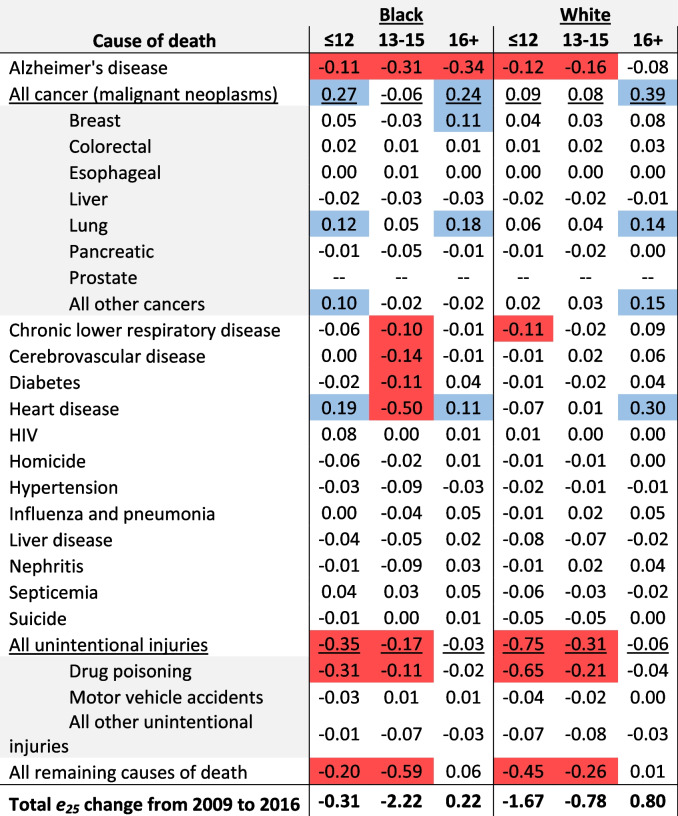
Contribution, in years, of specific causes of death to change in life expectancy from 2009 to 2016 among Black and white females in the Great Lakes region by education

Values represent the contribution, in years, to change in life expectancy from 2009 to 2016. Negative values reflect life expectancy losses over time. Conversely, positive values reflect life expectancy gains over time. Cells in red represent contributions less than or equal to -0.10 and cells in blue represent contributions greater than or equal to 0.10

Similar to white males, drug poisoning was the main cause of declining longevity among white females, but especially among those with ≤ 12 (0.65 years) and 13–15 years of education (0.21 years). Alzheimer’s disease was responsible for longevity declines among white females with ≤ 12 (0.12 years), 13–15 (0.16 years), and 16 + (0.08 years) years of education.

### Aim 3: Identify where in the life course each cause of death contributed most to life expectancy change

**Fig. 4:**
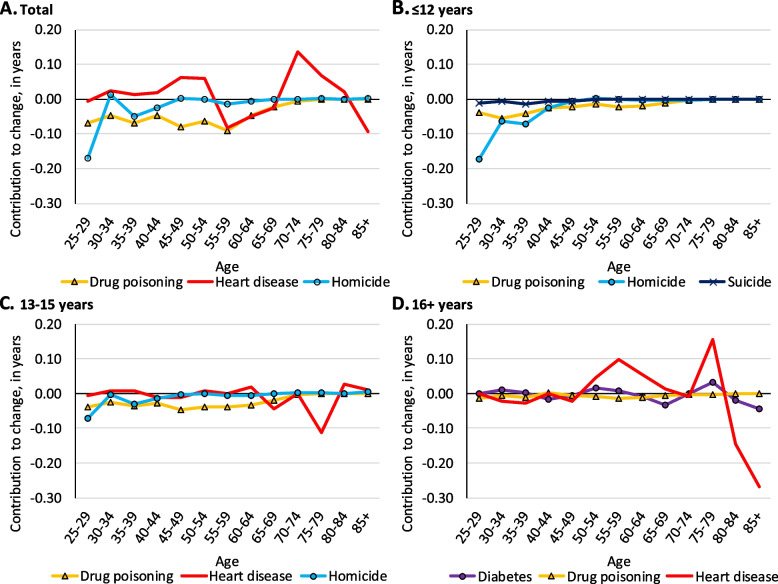
Age-specific contributions of select causes of death to change in life expectancy among Black males from 2009 to 2016 in the Great Lakes region by education

For ease of interpretation, we only included major leading contributors to life expectancy change by educational attainment in Figs. 4 through 7. Complete age- and cause-decomposition tables presenting the contribution of each cause of death for each age group are available in the additional files (see Additional file 2 – Additional file 13).

Among all Black males, drug poisoning made notable contributions to life expectancy losses before age 65, homicide contributed to losses before age 45, and heart disease contributed to losses at ages 55–69 and after age 80 (see Fig. 4, panel A). Homicide among Black males with ≤ 12 years of education made the largest contribution to longevity decline from ages 25–39, where it contributed 0.31 years (see Fig. 4, panel B). Among Black males with 13–15 years of education, drug poisoning contributed 0.15 years to longevity decline from ages 45–64 (see Fig. 4, panel C). Heart disease among Black males with 13–15 years of education made a notable 0.11-year contribution to longevity decline at ages 75–79. Black males with 16 + years of education gained > 0.10 year of longevity due to improvements in heart disease at ages 55–59, and ages 75–79, but lost 0.41 years from heart disease after age 80 (see Fig. 4, panel D).

**Fig. 5:**
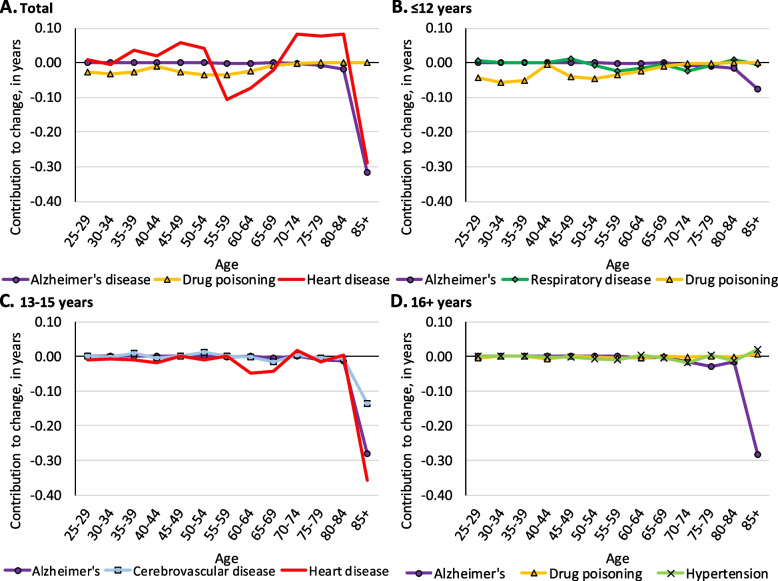
Age-specific contributions of select causes of death to change in life expectancy among Black females from 2009 to 2016 in the Great Lakes region by education

Among all Black females, heart disease contributed to longevity declines at ages 50–69 and after age 80, drug poisoning contributed to losses at ages 25–64, and Alzheimer’s disease contributed to losses after age 80 (see Fig. 5, panel A). Among Black females with ≤ 12 years of education, drug poisoning contributed 0.15 years to longevity decline between ages 25–39, and another 0.12-year decline between ages 45–59 (see Fig. 5, panel B). Black females with 13–15 years of education saw their life expectancy decline most from heart disease and Alzheimer’s disease at age 85 + , where they contributed 0.36 and 0.28 years, respectively (see Fig. 5, panel C). Alzheimer’s disease also made a notable 0.29-year contribution to longevity decline at age 85 + among Black females with 16 + years of education (see Fig. 5, panel D).

**Fig. 6:**
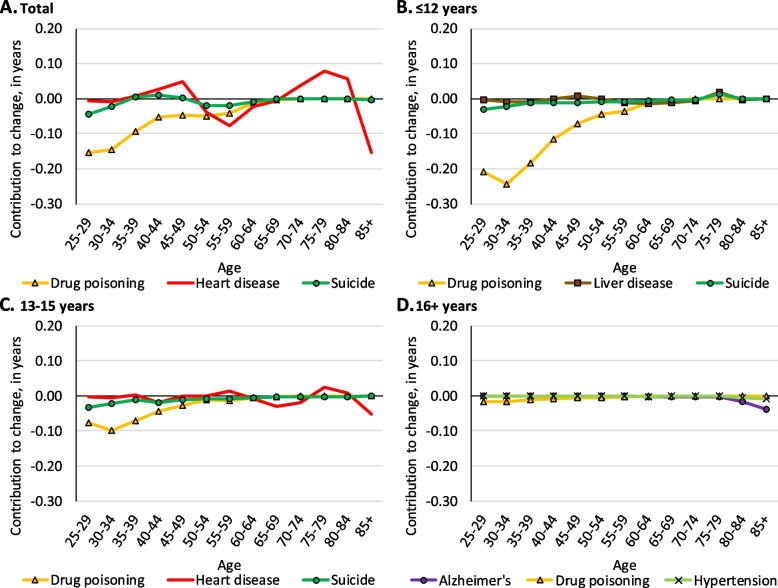
Age-specific contributions of select causes of death to change in life expectancy among white males from 2009 to 2016 in the Great Lakes region by education

Overall, among white males, drug poisoning made notable contributions to declining longevity before age 65 and heart disease contributed to losses at ages 50–64 and after age 80 (see Fig. 6, panel A). Drug poisoning among white males with ≤ 12 years of education made a considerable 0.80-year contribution to longevity decline before the age of 50 (see Fig. 6, panel B). The contribution of drug poisoning was also evident among white males with 13–15 years of education, where it contributed 0.31 years to longevity decline before age 50 (see Fig. 6, panel C).

**Fig. 7:**
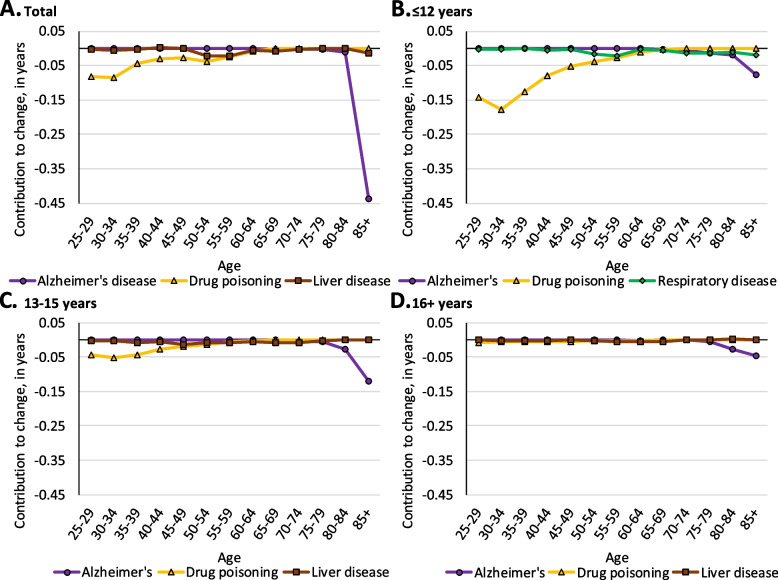
Age-specific contributions of select causes of death to change in life expectancy among white females from 2009 to 2016 in the Great Lakes region by education

Among all white females, drug poisoning made notable contributions to longevity losses before age 60 and Alzheimer’s disease contributed substantially to losses after age 80 (see Fig. 7, panel A). Similar to males, drug poisoning among females with ≤ 12 years of education was responsible for longevity declines at relatively young ages, contributing 0.57 years to longevity decline before age 50 (see Fig. 7, panel B). Among white females with 13–15 years of education, drug poisoning made smaller, yet notable contributions to longevity declines before age 40. Alzheimer’s disease among those with 13–15 years of education contributed 0.12 years to longevity decline at age 85 + (see Fig. 7, panel C). Among white females with 16 + years of education, Alzheimer’s disease contributed a small 0.08-year longevity decline after age 80 (see Fig. 7, panel D).

## Discussion

This investigation found that all groups in the Great Lakes region with < 16 years of education experienced a decline in life expectancy over the study period. Life expectancy declined most among Black females with 13–15 years of education, by more than two years. Among white males and females with ≤ 12 years of education and white males with 13–15 years of education, life expectancy declined by more than one year. Black males of all education levels, white females with 13–15 years of education, and Black females with ≤ 12 years of education all experienced longevity declines of less than one year. Homicide, which was especially problematic from ages 25–39, was the leading contributor to longevity declines among Black males with ≤ 12 years of education. Drug poisoning was the leading contributor to declining life expectancy among all other groups with ≤ 12 years of education; it was also a major contributor to longevity declines among Black males and white males and females with 13–15 years of education. Lastly, mortality from Alzheimer’s disease beyond age 80 limited gains in life expectancy among Black and white females with 16 + years of education.

Drug poisoning was a leading contributor to life expectancy decline among all sex, race, and education groups. However, the contribution of drug poisoning to longevity declines was greatest among those with < 16 years of education; life expectancy declines from drug poisoning were most notable among white males and females with a high school education or less. These findings suggest that this is a particularly vulnerable group that would benefit from public health efforts to reduce the risk of drug poisoning.

Rising drug poisoning mortality has been driven by the opioid epidemic in the U.S. [38, 39]. Between 2015 and 2017, there were more than 9,700 drug poisoning deaths among white males in the Great Lakes region – a mortality rate of 18.8 deaths per 100,000 people, which was six deaths per 100,000 above the drug poisoning mortality rate among white males in the rest of the U.S. [40]. The drug poisoning mortality rate among white females in the Great Lakes region was 7.2 deaths per 100,000 – nearly three deaths greater than the drug poisoning mortality rate among white females elsewhere in the U.S.. In addition, the drug poisoning mortality rate among Black males in the Great Lakes region was nearly 30 deaths per 100,000 – 17 deaths greater than the drug poisoning mortality rate among Black males in the rest of the U.S. Black females in the Great Lakes region faced a drug poisoning mortality rate of nearly 10 deaths per 100,000 people – six deaths more than the drug poisoning mortality rates among Black females in other parts of the U.S. Although drug poisoning has exacted a heavy toll on the longevity of white males and females without a college education, its disproportionate impact on Black communities in the Great Lakes region is clearly evident.

Research shows that robust state-level prescription drug monitoring programs could be effective in reducing nearly 20% of opioid overdose deaths [41]. More robust programs could monitor opioid prescriptions for patients and help prevent abuse and misuse that has commonly led to overdose mortality. Other strategies to reduce the impact of the opioid epidemic include education campaigns about the risks of opioids for patients, making overdose-preventing medication like Naloxone more widely accessible, and limiting supply of opioids on the market through more stringent regulation and restriction of pharmaceutical products [42].

Homicide between ages 25–39 was a major contributor to life expectancy decline among Black males with ≤ 12 years of education. The risk of homicide is greater for Black males residing in the Great Lakes region than their counterparts in the rest of the U.S. From 2015 to 2017, the homicide mortality rate among Black males in the Great Lakes region was 61 deaths per 100,000 people, compared to 37.6 deaths per 100,000 people among Black males in other parts of the U.S. [43]. The highest homicide mortality rate in the nation is among Black males in Illinois, where there were 74 deaths per 100,000 people from 2015 to 2017.

The large contribution of homicide to declining longevity among young, low-educated Black males in the Great Lakes region underscores the public health need to address structural factors influencing firearm homicide and reduce violence among young Black males. Communities of color have been disproportionately impacted by gun violence [44]. Major contributors to gun violence include structural racism and racial segregation, income inequality, and neighborhood and individual socioeconomic disadvantages [44–46]. Public health efforts must address structural inequities that underlie gun violence among Black males by actively engaging with the community to address the social determinants of gun violence and providing comprehensive resources and interventions to support communities at risk [47]. In addition to these structural efforts, it is important to engage at-risk youth and children to deter violent behavior, improve emotional intelligence, and reduce the chances of risky lifestyles in adulthood. The CDC offers recommendations to curb youth violence including programs to strengthen parenting skills and family relationships, provide quality education, mentor youth in after-school programs, and reach out to at-risk communities [48].

In addition to drug poisoning, heart disease among Black females and Alzheimer’s disease among both Black and white females were key contributors to longevity declines. The contribution of Alzheimer’s disease was particularly noteworthy among Black females with 13–15 and 16 + years of education. Conditions such as high blood pressure, diabetes, and high cholesterol increase the risk of developing both heart disease and Alzheimer’s disease. Existing research suggests that up to 80% of people with Alzheimer’s disease may also suffer from heart disease [49]. Regular exercise to increase blood and oxygen to the brain, maintaining a healthy diet, and keeping intellectually active at middle- and late-stages of life can help reduce the risk of developing Alzheimer’s disease in older age [49]. Targeted public health campaigns explaining risk-factors associated with Alzheimer’s disease could prove helpful in reducing risk of the disease among Black and white females.

There were some notable limitations in this investigation. There are concerns over the accuracy of educational attainment reported on death certificates, as they are completed by next of kin who may inaccurately report the education of the deceased. Previous studies have found that it is common to misclassify an individual that did not complete four years of high school as having completed four years of high school [50, 51]. In contrast, Black and Hispanic individuals who completed four years of high school are more likely than other race/ethnic groups to have their education underreported as not completing four years of high school [50]. Additionally, some evidence suggests that those with some college education but no Bachelor’s degree have been reported as having completed four years of college on their death certificate [50–52]. This is problematic as inaccuracies in education reporting on death certificates could distort racial longevity disparities across educational attainment. Future research of a smaller population or geographical area could benefit by adjusting for differential education reporting, which could improve mortality estimates for different education groups [50].

Future research could also benefit by accounting for immigrant populations when estimating life expectancy. The current analysis neither excluded nor stratified the population by immigrant status. Black immigrants, for instance, have been found to live nearly 8 years longer than their U.S.-born counterparts [53]. Considering that Black immigrants make up only 3.5% of all Black people residing in the Great Lakes region [54], it is unlikely that the findings of this investigation have been substantially influenced by the longevity patterns of immigrant populations in the region. In addition, future analyses could benefit from disaggregating the Great Lakes region into individual states to better identify impacted subgroups and create targeted interventions through state-level legislation and public health programs. Lastly, the COVID-19 pandemic disproportionately impacted Black males and females in the U.S. Early projections suggest that mortality from COVID-19 among Black people has reduced life expectancy by more than two years, widening the national Black-white life expectancy gap by more than 40% and erasing more than a decade of progress towards parity in longevity [55]. Future research incorporating the contribution of COVID-19 to longevity change among Black and white males and females in the Great Lakes region and nationally would likely reveal a new leading contributor to changes in life expectancy.

Despite these limitations, this investigation has several strengths. The use of restricted, uncensored death counts eliminated the need to employ imputation techniques to estimate life expectancy, as other studies have done [19, 22]. Measuring the contribution of specific causes of death, such as Alzheimer’s disease, drug poisoning, and homicide revealed important contributors to longevity decline that have been concealed in broad cause-of-death categories used in previous studies [12]. Additionally, measuring contributors across different age groups produced new evidence as to *where* along the life course causes of death are contributing most to longevity declines. These findings can be used to inform public health stakeholders of target demographic groups to implement interventions to address declining life expectancy and ongoing longevity disparities in the Great Lakes region.

## Conclusion

This investigation provided new evidence explaining longevity change in the Great Lakes region across different sex, race, and education groups that until now was unavailable. Findings for the Great Lakes region may have implications for other parts of the U.S. as well, where life expectancy has stalled or declined, and where longevity disparities persist across different populations. This study identified drug poisoning as a major contributor to declining life expectancy in the Great Lakes region, especially among Black males and white males and females with ≤ 12 years of education. Homicide among Black males with < 16 years of education was another key driver of longevity decline. Additionally, Alzheimer’s disease contributed to longevity declines after age 80, especially among Black females with 13–15 and 16 + years of education, and white females with 13–15 years of education. Public health stakeholders could address declining life expectancy in the Great Lakes region by implementing programs aimed at reducing drug poisoning mortality, especially among those with < 16 years of education, reducing violence and homicide among Black males with ≤ 12 years of education under the age of 40, and promoting healthy lifestyles at middle and older ages to reduce the risk of Alzheimer’s disease among all females.

## Supplementary Information

**Additional file 1. **Cause of death coding scheme.

**Additional file 2. **Age- and cause-specific contributors to change in life expectancy from 2009 to 2016 among Black males with ≤12 years in the Great Lakes region.

**Additional file 3. **Age- and cause-specific contributors to change in life expectancy from 2009 to 2016 among Black females with ≤12 years in the Great Lakes region.

**Additional file 4. **Age- and cause-specific contributors to change in life expectancy from 2009 to 2016 among Black males with 13-15 years in the Great Lakes region.

**Additional file 5. **Age- and cause-specific contributors to change in life expectancy from 2009 to 2016 among Black females with 13-15 years in the Great Lakes region.

**Additional file 6. **Age- and cause-specific contributors to change in life expectancy from 2009 to 2016 among Black males with 16+ years in the Great Lakes region.

**Additional file 7. **Age- and cause-specific contributors to change in life expectancy from 2009 to 2016 among Black females with 16+ years in the Great Lakes region.

**Additional file 8. **Age- and cause-specific contributors to change in life expectancy from 2009 to 2016 among white males with ≤12 years in the Great Lakes region.

**Additional file 9. **Age- and cause-specific contributors to change in life expectancy from 2009 to 2016 among white females with ≤12 years in the Great Lakes region.

**Additional file 10. **Age- and cause-specific contributors to change in life expectancy from 2009 to 2016 among white males with 13-15 years in the Great Lakes region.

**Additional file 11. **Age- and cause-specific contributors to change in life expectancy from 2009 to 2016 among white females with 13-15 years in the Great Lakes region.

**Additional file 12. **Age- and cause-specific contributors to change in life expectancy from 2009 to 2016 among white males with 16+ years in the Great Lakes region.

**Additional file 13. **Age- and cause-specific contributors to change in life expectancy from 2009 to 2016 among white females with 16+ years in the Great Lakes region.

## Data Availability

The data that support the findings of this study are available from U.S. National Center for Health Statistics but restrictions apply to the availability of these data, which were used under license for the current study, and so are not publicly available. Data are however available from the corresponding author upon reasonable request and with permission of U.S. National Center for Health Statistics.

## Abbreviations

U.S: United States
NCHS: National Center for Health Statistics
ICD: International Classification of Disease
ACS: American Community Survey
