# Supplementary material for: Declining life expectancy in the Great Lakes region: contributors to Black and white longevity change across educational attainment
Source: BMC Public Health. 2023 Apr 26;23:769. doi: 10.1186/s12889-023-15668-x (PMC10130305; doi:10.1186/s12889-023-15668-x)
Supplement: Supplementary file 1 — Additional file 1. Cause of death coding scheme. [file 12889_2023_15668_MOESM1_ESM.pdf]

Additional file 1. Cause of death coding scheme

Cause of death	358 Recode values
Alzheimer's disease	189
All cancer (malignant neoplasms)	
Breast cancer	104
Colorectal cancer	081-083
Esophageal cancer	77
Liver cancer	085-086
Lung cancer	93
Pancreatic cancer	88
Prostate cancer	113
All Other Cancers	072-075; 079; 087; 089; 091-092; 094-096; 098-099; 101-103; 106-111; 114-115; 117-120; 122-124; 125; 127; 129-132; 134-137; 139-140; 142-146
Cerebrovascular Disease	235-239
Diabetes	159
Heart Disease	199; 201-204; 207; 209; 211-215; 218-219; 221-222; 224-228; 230-233
HIV	049-053
Homicide	433-441
Hypertension	206; 208
Influenza and pneumonia	253; 255-257
Liver disease	298-302
Nephritis	323-325; 327; 329
Respiratory disease	264-269
Septicemia	23
Suicide	425-431
All unintentional injuries	
Drug poisoning	420
Motor vehicle accidents	386-388; 390-398
All other unintentional injuries	384; 399-402; 404-418; 421-423
All other causes	All else
