# Supplementary material for: Declining life expectancy in the Great Lakes region: contributors to Black and white longevity change across educational attainment
Source: BMC Public Health. 2023 Apr 26;23:769. doi: 10.1186/s12889-023-15668-x (PMC10130305; doi:10.1186/s12889-023-15668-x)
Supplement: Supplementary file 5 — Additional file 5. Age- and cause-specific contributors to change in life expectancy from 2009 to 2016 among Black females with 13-15 years in the Great Lakes region. [file 12889_2023_15668_MOESM5_ESM.pdf]

Additional file 5. Age- and cause-specific contributors to change in life expectancy from 2009 to 2016 among Black females with 13-15 years in the Great Lakes region

Age	25-29	30-34	35-39	40-44	45-49	50-54	55-59	60-64	65-69	70-74	75-79	80-84	85+	Total
Alzheimer's disease	0.00	0.00	0.00	0.00	0.00	0.00	0.00	0.00	0.00	0.00	-0.01	-0.01	-0.28	-0.31
Breast Cancer	0.00	0.00	0.00	0.00	0.00	0.01	0.01	0.02	-0.01	0.01	-0.01	0.00	-0.07	-0.03
Colorectal cancer	0.00	0.00	0.00	0.01	-0.01	0.00	0.01	0.00	0.01	0.00	0.01	0.00	-0.01	0.01
Esophageal cancer	0.00	0.00	0.00	0.00	0.00	0.00	0.00	0.00	0.00	0.00	0.00	0.00	0.00	0.01
Liver cancer	0.00	0.00	0.00	0.00	0.00	0.00	0.00	-0.01	-0.01	0.00	0.00	0.00	-0.01	-0.03
Lung cancer	0.00	0.00	0.00	0.01	0.01	0.02	0.00	0.01	0.00	0.00	0.04	-0.01	-0.03	0.05
Pancreatic cancer	0.00	0.00	0.00	0.00	0.00	0.00	0.00	0.00	-0.01	0.01	-0.01	-0.01	-0.02	-0.05
Prostate cancer	0.00	0.00	0.00	0.00	0.00	0.00	0.00	0.00	0.00	0.00	0.00	0.00	0.00	0.00
All other cancer	-0.01	-0.01	0.01	0.01	0.01	0.00	0.00	0.01	-0.01	-0.01	-0.02	0.01	-0.01	-0.02
Cerebrovascular disease	0.00	0.00	0.00	0.00	0.00	-0.01	-0.01	-0.01	-0.01	0.00	-0.03	0.00	-0.02	-0.10
Diabetes	0.00	0.00	0.01	0.00	0.00	0.01	0.00	0.00	-0.01	0.00	0.00	-0.01	-0.14	-0.14
Heart disease	0.00	0.00	-0.01	-0.01	-0.02	-0.02	0.00	-0.01	-0.01	0.00	0.00	-0.01	-0.03	-0.11
HIV	-0.01	-0.01	-0.01	-0.02	0.00	-0.01	0.00	-0.05	-0.04	0.02	-0.02	0.00	-0.36	-0.50
Homicide	0.00	0.00	0.00	0.00	0.00	0.00	0.00	0.00	0.00	0.00	0.00	0.00	0.00	0.00
Hypertension	-0.01	-0.01	0.00	0.00	0.00	0.00	0.00	0.00	0.00	0.00	0.00	0.00	0.00	-0.02
Influenza / pneumonia	0.00	0.00	0.00	0.00	0.00	0.00	0.00	0.00	-0.01	0.00	-0.01	0.00	-0.08	-0.09
Liver disease	0.00	-0.01	0.01	0.00	0.00	0.00	0.00	-0.01	-0.01	0.01	0.00	0.00	-0.03	-0.04
Nephritis	0.00	0.00	0.00	0.00	0.00	0.00	0.00	0.00	0.00	0.00	0.00	0.00	-0.02	-0.05
Respiratory	0.00	0.00	0.00	0.00	0.00	0.00	0.00	0.00	-0.01	-0.01	0.00	-0.01	-0.03	-0.09
Septicemia	0.00	0.00	0.00	0.00	0.00	0.00	0.00	0.00	0.02	0.00	0.00	0.00	0.01	0.03
Suicide	-0.01	0.00	0.00	0.00	0.00	0.00	0.00	0.00	0.00	0.00	0.00	0.00	0.00	0.00
Drug poisoning	-0.02	-0.02	-0.02	-0.01	-0.01	-0.01	-0.01	-0.02	0.00	0.00	0.00	0.00	0.00	-0.11
Motor vehicle accidents	0.01	-0.01	0.00	0.00	0.00	0.00	0.00	0.00	0.00	0.00	0.00	0.00	0.00	0.01
All other unintentional	0.00	0.00	0.00	-0.01	0.00	0.00	0.00	-0.01	0.00	-0.01	0.00	0.00	-0.02	-0.07
All remaining causes	0.01	-0.02	-0.01	0.00	0.00	-0.02	0.00	-0.04	-0.03	0.00	-0.01	-0.08	-0.38	-0.59
