# Supplementary material for: Declining life expectancy in the Great Lakes region: contributors to Black and white longevity change across educational attainment
Source: BMC Public Health. 2023 Apr 26;23:769. doi: 10.1186/s12889-023-15668-x (PMC10130305; doi:10.1186/s12889-023-15668-x)
Supplement: Supplementary file 6 — Additional file 6. Age- and cause-specific contributors to change in life expectancy from 2009 to 2016 among Black males with 16+ years in the Great Lakes region. [file 12889_2023_15668_MOESM6_ESM.pdf]

Additional file 6. Age- and cause-specific contributors to change in life expectancy from 2009 to 2016 among Black males with 16+ years in the Great Lakes region

Age	25-29	30-34	35-39	40-44	45-49	50-54	55-59	60-64	65-69	70-74	75-79	80-84	85+	Total
Alzheimer's disease	0.00	0.00	0.00	0.00	0.00	0.00	0.00	-0.01	-0.01	-0.01	0.03	-0.02	0.00	-0.02
Breast Cancer	0.00	0.00	0.00	0.00	0.00	0.00	0.00	0.00	0.00	0.00	0.00	0.00	0.00	0.00
Colorectal cancer	0.00	-0.01	0.00	0.00	-0.01	-0.02	0.02	0.02	0.01	0.01	0.01	0.00	0.01	0.04
Esophageal cancer	0.00	0.00	0.00	0.00	0.00	0.00	0.00	-0.01	0.00	0.00	0.00	0.00	0.02	0.02
Liver cancer	0.00	0.00	0.00	0.01	0.00	0.00	0.01	-0.02	-0.01	-0.01	0.00	0.00	-0.03	-0.03
Lung cancer	0.00	0.00	0.00	0.00	0.00	0.00	0.03	0.04	0.01	0.01	0.05	0.00	0.04	0.17
Pancreatic cancer	0.00	0.00	0.00	0.00	0.00	0.00	0.01	0.00	0.00	0.01	0.04	0.00	0.01	0.07
Prostate cancer	0.00	0.00	0.00	0.00	0.00	-0.01	-0.01	-0.02	-0.02	-0.01	0.05	-0.01	0.01	-0.01
All other cancer	0.01	-0.01	0.00	0.01	-0.01	-0.01	0.03	-0.02	0.00	0.04	0.04	-0.06	-0.01	0.02
Cerebrovascular disease	0.00	0.00	0.00	0.00	0.00	0.00	0.02	0.00	0.02	0.00	-0.02	-0.02	-0.01	-0.03
Diabetes	0.00	0.00	0.00	0.00	0.00	0.00	0.03	0.01	0.00	0.00	0.02	-0.04	-0.01	0.00
Heart disease	0.00	0.01	0.00	-0.02	-0.01	0.01	0.01	-0.01	-0.03	0.00	0.03	-0.02	-0.04	-0.06
HIV	0.00	-0.02	-0.03	0.00	-0.02	0.05	0.10	0.05	0.01	-0.01	0.16	-0.15	-0.27	-0.12
Homicide	0.00	0.02	0.01	0.01	0.03	0.02	0.00	0.00	0.01	0.00	0.00	0.00	0.00	0.10
Hypertension	0.00	-0.03	0.01	0.02	0.01	-0.01	0.01	0.00	0.00	0.00	0.00	0.00	0.00	0.01
Influenza / pneumonia	0.00	0.00	0.00	0.00	0.00	0.00	0.00	0.01	0.00	-0.02	0.01	-0.01	0.00	-0.02
Liver disease	0.00	0.00	0.00	0.00	0.00	0.01	0.01	0.00	0.00	-0.02	0.02	-0.02	0.01	0.01
Nephritis	0.00	0.00	0.00	0.00	0.00	0.00	0.03	0.00	0.00	-0.02	0.00	-0.01	0.01	0.00
Respiratory	0.00	0.01	0.00	0.00	0.00	0.01	0.01	0.00	-0.01	-0.03	0.03	-0.01	-0.03	-0.04
Septicemia	0.00	0.00	0.01	-0.01	-0.01	0.01	0.01	0.02	0.00	-0.01	0.00	-0.02	0.01	0.02
Suicide	0.01	-0.01	0.01	0.00	0.00	0.00	-0.01	0.00	0.00	0.00	0.00	0.00	-0.01	-0.01
Drug poisoning	-0.01	-0.01	-0.01	0.00	-0.01	-0.01	-0.01	-0.01	0.00	0.00	0.00	0.00	0.00	-0.08
Motor vehicle accidents	0.00	0.01	0.01	0.00	-0.01	0.01	0.01	0.00	0.00	0.00	0.01	0.00	-0.01	0.04
All other unintentional	-0.02	-0.01	-0.01	0.00	0.00	0.00	0.00	0.00	-0.01	0.00	0.00	-0.01	0.00	-0.06
All remaining causes	-0.01	-0.02	0.00	0.00	0.01	0.02	0.05	0.00	-0.06	-0.06	0.05	-0.12	-0.20	-0.33
