# Supplementary material for: Declining life expectancy in the Great Lakes region: contributors to Black and white longevity change across educational attainment
Source: BMC Public Health. 2023 Apr 26;23:769. doi: 10.1186/s12889-023-15668-x (PMC10130305; doi:10.1186/s12889-023-15668-x)
Supplement: Supplementary file 8 — Additional file 8. Age- and cause-specific contributors to change in life expectancy from 2009 to 2016 among white males with ≤12 years in the Great Lakes region. [file 12889_2023_15668_MOESM8_ESM.pdf]

Additional file 8. Age- and cause-specific contributors to change in life expectancy from 2009 to 2016 among white males with ≤12 years in the Great Lakes region

| Age | 25-29 | 30-34 | 35-39 | 40-44 | 45-49 | 50-54 | 55-59 | 60-64 | 65-69 | 70-74 | 75-79 | 80-84 | 85+ | Total |
|---|---|---|---|---|---|---|---|---|---|---|---|---|---|---|
| Alzheimer's disease | 0.00 | 0.00 | 0.00 | 0.00 | 0.00 | 0.00 | 0.00 | 0.00 | 0.00 | 0.00 | 0.02 | 0.00 | -0.01 | 0.00 |
| Breast Cancer | 0.00 | 0.00 | 0.00 | 0.00 | 0.00 | 0.00 | 0.00 | 0.00 | 0.00 | 0.00 | 0.00 | 0.00 | 0.00 | 0.00 |
| Colorectal cancer | 0.00 | 0.00 | 0.00 | 0.00 | 0.00 | 0.00 | 0.00 | 0.00 | 0.00 | 0.00 | -0.04 | 0.00 | 0.00 | -0.02 |
| Esophageal cancer | 0.00 | 0.00 | 0.00 | 0.00 | 0.00 | 0.00 | 0.00 | 0.00 | -0.01 | 0.00 | 0.01 | 0.00 | 0.00 | 0.01 |
| Liver cancer | 0.00 | 0.00 | 0.00 | 0.00 | 0.00 | 0.01 | 0.00 | -0.01 | -0.01 | 0.00 | 0.02 | 0.00 | 0.00 | 0.00 |
| Lung cancer | 0.00 | 0.00 | 0.00 | 0.01 | 0.01 | 0.01 | 0.02 | 0.03 | 0.04 | 0.02 | -0.14 | 0.01 | 0.00 | 0.02 |
| Pancreatic cancer | 0.00 | 0.00 | 0.00 | 0.00 | 0.00 | 0.00 | 0.00 | 0.00 | 0.00 | 0.00 | 0.01 | 0.00 | 0.00 | 0.00 |
| Prostate cancer | 0.00 | 0.00 | 0.00 | 0.00 | 0.00 | 0.00 | 0.00 | 0.00 | 0.00 | 0.00 | -0.03 | 0.00 | 0.01 | -0.02 |
| All other cancer | 0.00 | 0.00 | 0.00 | 0.00 | 0.01 | 0.01 | 0.01 | 0.01 | 0.00 | 0.00 | -0.04 | 0.00 | 0.00 | -0.01 |
| Cerebrovascular disease | 0.00 | 0.00 | 0.00 | 0.00 | 0.00 | 0.00 | 0.00 | 0.00 | -0.01 | -0.01 | -0.01 | 0.01 | 0.00 | -0.03 |
| Diabetes | 0.00 | 0.00 | 0.00 | 0.00 | 0.00 | 0.00 | 0.00 | 0.00 | 0.00 | 0.00 | -0.04 | 0.00 | 0.01 | -0.03 |
| Heart disease | 0.00 | 0.00 | 0.00 | -0.01 | -0.01 | 0.00 | 0.00 | 0.00 | 0.00 | -0.01 | 0.02 | 0.00 | 0.00 | -0.01 |
| HIV | 0.00 | -0.01 | 0.00 | 0.00 | 0.00 | 0.00 | 0.02 | 0.01 | -0.03 | -0.02 | -0.02 | 0.02 | 0.02 | -0.02 |
| Homicide | 0.00 | 0.00 | 0.00 | 0.00 | 0.00 | 0.00 | 0.00 | 0.00 | 0.00 | 0.00 | 0.00 | 0.00 | 0.00 | 0.01 |
| Hypertension | 0.00 | -0.01 | 0.00 | 0.00 | 0.00 | 0.00 | 0.00 | 0.00 | 0.00 | 0.00 | 0.00 | 0.00 | 0.00 | -0.02 |
| Influenza / pneumonia | 0.00 | 0.00 | 0.00 | 0.00 | 0.00 | 0.00 | 0.00 | 0.00 | 0.00 | 0.00 | 0.01 | 0.00 | 0.00 | 0.00 |
| Liver disease | 0.00 | 0.00 | 0.00 | 0.00 | 0.00 | 0.00 | 0.00 | 0.00 | 0.00 | 0.00 | -0.01 | 0.00 | 0.01 | -0.02 |
| Nephritis | 0.00 | -0.01 | -0.01 | 0.00 | 0.01 | 0.00 | -0.01 | -0.01 | -0.01 | -0.01 | 0.02 | 0.00 | 0.00 | -0.04 |
| Respiratory | 0.00 | 0.00 | 0.00 | 0.00 | 0.00 | 0.00 | 0.00 | 0.00 | 0.00 | 0.00 | -0.02 | 0.00 | 0.01 | -0.02 |
| Septicemia | 0.00 | 0.00 | 0.00 | 0.00 | 0.00 | 0.00 | 0.00 | 0.00 | 0.00 | 0.00 | 0.02 | 0.00 | 0.00 | -0.01 |
| Suicide | -0.03 | -0.02 | -0.01 | -0.01 | -0.01 | -0.01 | -0.01 | -0.01 | 0.00 | 0.00 | 0.01 | 0.00 | 0.00 | -0.10 |
| Drug poisoning | -0.21 | -0.24 | -0.18 | -0.11 | -0.07 | -0.04 | -0.03 | -0.01 | -0.01 | 0.00 | 0.00 | 0.00 | 0.00 | -0.92 |
| Motor vehicle accidents | -0.01 | 0.00 | -0.01 | 0.00 | 0.00 | -0.01 | 0.00 | 0.00 | 0.00 | 0.00 | 0.01 | 0.00 | 0.00 | -0.03 |
| All other unintentional | 0.00 | -0.01 | 0.00 | 0.00 | 0.01 | 0.00 | -0.01 | 0.00 | 0.00 | 0.00 | 0.03 | 0.00 | -0.01 | -0.01 |
| All remaining causes | -0.02 | -0.02 | -0.01 | 0.00 | -0.01 | -0.01 | 0.00 | -0.03 | -0.04 | -0.03 | 0.17 | -0.01 | -0.02 | -0.04 |
